# Racial Disparities and the Use of Technology for Self-Management in Blacks with Heart Failure: A Literature Review

**DOI:** 10.1007/s11897-014-0213-9

**Published:** 2014-07-11

**Authors:** Hannah Anderson Hughes, Bradi B. Granger

**Affiliations:** Duke University School of Nursing, 307 Trent Drive, Durham, NC 27710 USA

**Keywords:** Heart failure, Self-management, Self-care, Technology, Text messaging, Telemedicine, e-Health, mHealth, Email, Internet, Web, Cell phone, Mobile technology, African American, Blacks, Patient education, Social networking, Social support

## Abstract

Heart failure is a debilitating illness that requires patients to be actively engaged in self-management. Self-management practices, including maintenance and management of an evidence-based medication regimen, are associated with improved outcomes. Yet, sustained engagement with self-management practices remains a challenge. Both self-management practices and clinical outcomes differ by race, with the poorest self-management and clinical outcomes reported in Blacks. Contemporary interventions to address self-management and reverse current trends in outcomes have evaluated the use of technology. Technological innovations, such as text messaging, social networking, and online learning platforms may provide a more accessible means for self-management of heart failure, yet these innovations have been understudied in the population at greatest risk – Blacks with heart failure. We conducted a review and discovered only four studies evaluating use of technology for self-management in Blacks. More studies are needed to close the gap on racial disparities and use of technology for self-management.

## Introduction

### Heart Failure Morbidity, Mortality, & Costs

Heart failure is a debilitating, chronic illness that affects 5.7 million people, and 55,000 patients die annually [1]. An estimated 400,000 to 700,000 new cases of heart failure are diagnosed each year [2]. Less than 50 % of patients live for 5 years after their initial diagnosis; less than 25 % are alive after 10 years [2]. Heart failure is most often associated with progressive decline and poor quality of life. Annual heart failure care costs are documented at $34.4 billion, but despite these staggering expenditures and the use of resources to address this trend, heart failure remains the leading cause of re-hospitalization for patients aged 65 years and older [3]. Each year more than one million patients are hospitalized for heart failure in the United States [4]; these hospitalizations are largely preventable [5]. The median 30-day re-hospitalization rate among heart failure patients has been reported to be as high as 24 % [6], and has been associated with poor self-management practices. The purpose of this review was to evaluate systematically the use of technology for self-management in patients with heart failure, and in particular, to evaluate the rigor of studies reporting racial disparity in use of technology-based strategies.

### Heart Failure Self-Management

Self-care is defined as “a naturalistic decision-making process by which individuals make choices about behaviors that maintain physiologic stability and the response to symptoms when they occur.” [7] Naturalistic decision-making involves rapidly making high-stakes decisions in dynamic, real-world situations that are often ambiguous and that involve ambiguity [8]. Riegel and Dickson differentiated self-care from self-management in heart failure patients, characterizing self-care as a process that involves three aspects: maintenance, management, and confidence [9]. Self-care maintenance involves following the advice of healthcare professionals regarding the symptom monitoring and treatment adherence behaviors necessary to promote physiological stability. Self-management is an active, deliberate, decision-making process that occurs in response to one’s symptoms. Confidence relates to the individual’s belief in his or her ability to self-manage heart failure, is a moderator of self-care management, and mediates the relationship between social support and effective heart failure self-care [9]. Vrijens and colleagues support the definitions proposed by Riegel and Dickson and describe self-management specifically as it related to medication-taking by defining the medication-taking aspect of self-care behavior as a distinct component of self-management [10••].

Self-care of heart failure also requires experience and skill, motivation, confidence, development of habits, adequate functional and cognitive abilities, social support, and access to care, and the goal of implementing adequate self-care is to promote stability, health, well-being, and quality of life [8]. Both the broad nature of self-care and the more focused aspect of self-management place similar requirements on patients: being proactive, adhering to professional guidance, paying close attention to one’s body signals, and developing appropriate coping behaviors [9]. However, self-management differs in that it involves patients making therapeutic adjustments; making decisions regarding their care that are usually reserved for healthcare professionals [11].

According to heart failure guidelines, self-management involves four aspects: medication management, symptom management, dietary modifications, and activity adjustments [12, 13]. These self-management activities are complex, requiring work on the patient’s part [14]; patients are often knowledgeable regarding self-management measures, but have difficulty implementing these activities [15]. Only one in 10 heart failure patients are expected to master self-management [16], yet patients who are actively engaged in self-management have better outcomes [8], less than half of the risk of all-cause mortality, hospitalization, or emergency room admission when compared to other less engaged patients [17]. Among all patients with heart failure, adoption of successful self-management strategies differs by race [18, 19••], with those at highest risk demonstrating the lowest rates of successful engagement with self-management strategies [20–24].

### Heart Failure in Blacks

Blacks are currently at an increased risk for developing heart failure and have a higher risk of its occurrence [3, 25••]. In patients less than 75 years of age, Blacks have the highest incidence of heart failure [25••] and often an earlier age of onset [26]. Blacks are expected to remain the group most affected by heart failure, and heart failure prevalence among Blacks is expected to increase by 29 % between 2012 and 2030 [3]. Blacks also have a higher incidence of co-morbidities and risk factors for development of heart failure, including hypertension and diabetes [3, 21, 25••], and Blacks are 50 % less likely to maintain their blood pressure under control. Additionally, Blacks with heart failure have poorer outcomes than Whites [25••]. Annual rates of new heart failure events per 1,000 population for Black men are 16.9 for those ages 65 to 74 years of age, 25.5 for those ages 75 – 84 years of age when compared to 15.2 White men ages 65 – 74 years of age and 31.7 in those ages 75 – 84 years old. In Black women, the rates of new events are 14.2 for those ages 65 to 74 years of age and 25.5 among those ages 75 – 84 years old when compared to White women, whose rates are 8.2 at ages 65 – 74 and 19.8 at ages 75 – 84. Heart failure before 50 years of age was more common in Blacks than Whites [25••]. In 2010, the overall death rate for heart failure was 84 per 1,000. Death rates among Black males was 101.7 per 1,000 when compared to White males, who were 99.9 per 1,000. White females had a death rate of 74.1 per 1,000 as compared to Black females with a rate of 79.1 per 1,000 [25••].

Hospitalizations for heart failure are more prevalent among Blacks and they have a higher mortality rate than Whites [21, 25••, 27]. Efforts to decrease heart failure morbidity and mortality have not been fully effective. Despite the various programs designed to promote primary and secondary prevention, none have been successful in halting the alarming prevalence of heart failure among Blacks and subsequent negative outcomes. Access to care issues remains a factor that contributes to disparities in this patient population [26, 28]. Effective strategies designed to improve heart failure self-care in Blacks might reduce, and possibly eliminate, racial disparities in heart failure.

### The Role of Culture

Social norms, cultural beliefs, and cultural preferences significantly influence self-care practices [19••, 29]. Becker characterized self-care in Blacks with chronic illnesses as a process that is deeply rooted in cultural beliefs [30]. Because self-management is an aspect of self-care, culture must be considered when attempting to improve self-management in a minority population. A culturally-based observation is that Blacks with heart failure use different coping mechanisms; they often delay seeking treatment and rely on spirituality and social support as coping mechanisms [18]. In a study of 30 Blacks with heart failure, Dickson and colleagues noted the association between culture and self-care. Self-care, on a whole, as measured by the SCHFI, was poor, with self-management having the lowest scores of all three sub-scales. Investigators concluded that culturally sensitive interventions should be designed and implemented [19••]. Three other international studies identified differences in self-care practices and beliefs among culturally-diverse patients [31–33]. Because Blacks with heart failure are affected at a disparate rate, their unique responses to technologically based interventions for self-care should be explored.

### Technological Innovations for Heart Failure Self-Management

Technology-based self-management solutions take many forms. As of January 2014, 90 % of adults have a cell phone, 58 % have a smartphone, and 42 % have a tablet computer [34]. Innovative strategies to promote self-management using mobile technologies are widely accessible, and yet are understudied. Cell phones reach the broadest audience. Cell phone ownership is high and almost equal amongst Whites, Blacks, and Hispanics (90 – 92 %). There is almost no difference in ownership of cell phones in urban, suburban, and rural areas (88 – 92 %); 87 % of adults with less education (high school diploma or less) own a cell phone, and 84 % of those who earn less than $30,000 per year have cell phones [34]. These data suggest that mobile technologies transcend racial, geographic, and educational barriers, and perhaps socioeconomic status as well.

The use of technology to promote symptom monitoring and self-management in heart failure is a not a new concept, yet efforts to implement effectively technologically based interventions are understudied. Thus far, findings suggest that remote monitoring of heart failure patients reduces all-cause mortality, heart failure related re-admissions, improves health-related quality of life, and may also reduce costs [35]. Technology-based medication management interventions, designed to remind patients to take medications, or to refill treatment regimen prescriptions have shown equivocal results [37]. Studies to assess the effectiveness of technology-based interventions are limited by non-randomized design, power, and sample size, and as a result, little is known regarding whether these interventions have resulted in better patient self-management, and subsequently, improved outcomes. In addition, although studies suggest racial differences in access and use of technology-based self-management strategies, the evidence to support these early observations is weak.

## Method

**Fig. 1:**
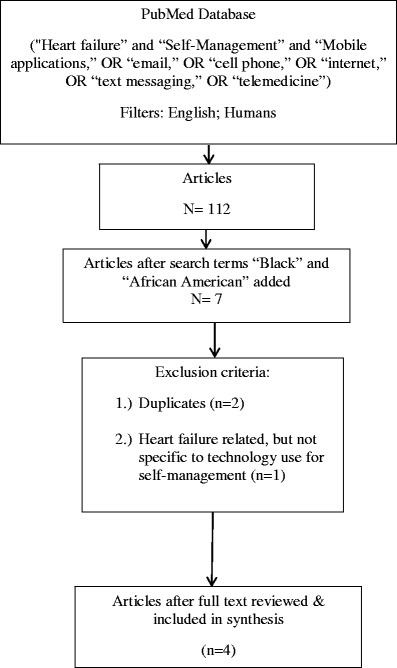
Search process flowchart

We searched the PubMed database using the initial search terms: “mobile applications,” “email,” “cell phone,” “internet,” “text messaging,” “telemedicine,” “self-management,” and “heart failure.” We filtered the articles by those involving human subjects and written in the English language. One hundred and twelve articles were initially found. We then added the search term “Black” to the initial search terms and yielded two articles. We subsequently added the search term “African American” to the initial search terms and yielded five articles. Two duplicates were removed. We screened the titles and abstracts for their relevance to the topic. One was omitted because it was unrelated to heart failure self-management using technology. We then screened the full text of the remaining four articles. Four articles are included in this review. Figure 1 describes the search process. The four articles were abstracted into a database using the matrix method [36]. Table 1 summarizes the detail of the studies included. Themes were identified by synthesizing the literature. The findings are organized based on the identified themes.

**Table 1:** Summary of articles

Author(s)	Study Design	Setting/Sample	Technology(ies) Used	Strengths/Weaknesses
Nahm, et al. (2008)	Exploratory, Single Group Survey Design 4-week intervention	N = 44; (21 or 47.7 % Black) Mean age = 72 years SD = 9 Convenience sample Recruited from a parent MCCD study over 5 months	Internet	Strengths: • Analysis conducted based on Black sample Weaknesses: • Lack of a control group • Lack of random sampling • Small sample size and Black sample • Short intervention length • Majority men (71.6 %) • Majority with NYHA Class III (83 %) • Researcher-developed measures without reliability/validity
Benatar, et al. (2010)	Prospective, randomized	N = 216 (186 or 86.1 % Black) Mean age = 63.06 years SD = 12.09 Convenience sample 3 home healthcare agencies & 2 medical centers	• Trans-telephonic monitoring devices • Internet • Telephone	Strengths: • Large sample size • RCT design with control group • 4 data collection time points • No attrition during the 3 month intervention • Four study settings • Large Black sample Weaknesses: Predominantly female sample (63 %)
Copeland, et al. (2010)	Prospective, randomized controlled trial 1-year intervention	N = 458 ( 32 or 7 % Black) Mean age = 70 years SD 11 (range 45 – 95) Convenience sample Veteran’s Affairs	• Telephone • EMR	Strengths: • RCT design with control group • AHA guidelines used to develop the educational content • Motivational interviewing & coaching used • Length of the intervention • Three data collection time points • Tailored intervention frequency based on categorization of patients as low, medium, or high risk Weaknesses: • Small Black sample • Heterogeneous VA sample • Baseline differences between the control and the intervention groups • Single location participant recruitment • No analysis on the Black sample
Nundy, et al. (2013)	Pre/Post Study 30-day intervention	N = 6 (100 % Black) N = 15 enrolled Mean age = 50 years Convenience sample Large, academic medical center	• Mobile Phone • SMS	Strengths: • 100 % Black sample • Assessment of self-care pre & post intervention Weaknesses: • Small sample size • Lack of random sampling. Single location participant recruitment

## Findings

To our knowledge, the literature related specifically to technology use for self-management in Blacks with heart failure is limited to four studies: one that investigates the use of an e-Health intervention [37], one a text messaging program [38••], one a telephone-based motivational interviewing and coaching program [39], and the fourth a transtelephonic monitoring program [40]. Four themes were identified from the synthesis of the four articles: (1) participants reported positive usability and acceptability of the technological intervention, (2) participants desired more instruction and training in using the technology, (3) participants viewed technology as a means of providing support, (4) technologically based interventions largely improved outcomes.

### Availability, Feasibility, Usability, & Acceptability

Heart failure patients were largely satisfied with and responded positively to the technologically based intervention [37, 38••, 39]. In the text messaging intervention, participants (n = 6) reported 100 % satisfaction with the mobile-based heart failure self-management program and said that they would recommend the program to a friend or family member [38••]; participants (n = 44) in the e-Health online management program reported high confidence with using telemonitoring devices (mean = 27.1, SD 18.9, range = 3-30) and with using web-based learning modules (mean = 7.6, SD = 3.2, range = 1-10), and stated that the program was easy to use [37]. There were no significant differences in the confidence for using telemonitoring devices and readiness to use the internet between the Black and White participants [37]. Lastly, the majority of participants also reported feeling comfortable with text messaging and had unlimited text messaging plans [38••].

### Desire for Additional Instructions and Training

Heart failure patients desired additional training to use technology effectively for self-management [37]. Fifty percent of the participants in the e-Health program study who were non-users of the internet (n = 17) indicated that they would be willing to learn to use the internet if training was provided [37], and some enrolled in the text messaging program requested additional training in texting [38••]. Assessing participants’ readiness to use technology may be an important baseline measurement for later comparison with study findings. Despite the lack of a baseline assessment, it seems evident that older Blacks with heart failure might be willing to use technology for self-management, if provided with adequate preparation and training.

### Technology Use as a Means of Support

Heart failure patients felt supported and cared for during their participation in the technologically-based interventions [37, 38••]. Participants felt that reminders sent via the text messaging program symbolized that someone cared enough to be concerned with how they took care of themselves [37]; those in the e-Health program felt confident in using telemonitoring because it meant that someone was watching and would alert them if something was wrong [37]. Technology-based interventions might not limit human connections, but rather, they might convey caring and concern for well-being because of the constant contact between patients and providers. A combination of technology-based reminders coupled with in-person communication has been shown to be most effective in promoting medication self-management [41••].

### Improved Outcomes

Technologically based interventions resulted in improved outcomes related to self-management, re-hospitalizations, costs, and quality of life for heart failure patients [37, 39, 40].

#### Improved Self-Management

Technologically based interventions improved heart failure patients’ overall and medication-specific self-management [37, 39]. In the text messaging program, both objective and subjective measurements suggest improvements in self-management following the text messaging program. Improvements in the mean scores on the Self-Care of Heart Failure Index (SCHFI) [42, 43] on all three sub-scales were noted between pre-intervention and post-intervention assessments, with mean self-care maintenance increasing from 49 to 78, self-care management increasing from 57 to 86, and self-care confidence increasing from 57 to 75. Specifically, improvement was seen in forgetting to take medicines (pre-intervention: 1.9 [SE 0.22] and post-intervention 1.3 [SE 0.52] *p* = 0.02), in addition to five other SCHFI measures: weighing self, eating a low-salt diet, avoiding getting sick, contacting the physician in the case of worsening symptoms, and confidence in evaluating symptoms. Five of these were included in the seven that were specifically targeted by the text messaging intervention. Improvements in self-care maintenance and management were clinically significant [38••]. Subjectively, participants in the text messaging intervention study reported that the intervention resulted in their improved self-management because it increased their disease awareness, reinforced the importance of self-management, included reminders, and provided feedback [38••].

#### Decrease in Re-hospitalization Rates

Patients who participated in the trans-telephonic monitoring study showed improved self-efficacy with medication use and were almost half as likely to be re-admitted compared to the patients in the control group (13 vs. 24; *p* ≤ 0.001) [36]. In subgroup analysis of readmissions at 3 months, patients who received ACEI and Beta Blockers were compared to those who did not. Patients in the telephonic intervention group who were on ACEI and Beta Blockers had lower readmission rates (11 % vs. 23 %; *p* = 0.003). Patients who were not prescribed either an ACEI or beta-blocker in both the intervention and control groups had a similar rate of re-admission (19 % and 25 %). When patients were admitted, inpatient length of stay in the intervention group was less than half of that of the control group (49.5 vs. 105.0 days; *p* ≤ 0.001). The outcome was sustainable at the 6- and 12-month marks, with 38 versus 63 readmissions (*p* ≤ 0.05) in the intervention group at 6 months and 75 versus 103 (*p* = 0.12) at 12 months [40].

#### Costs

Benatar and colleagues found that costs were significantly reduced in the patients who participated in the intervention group. At 3 months, readmission charges were $65,023 when compared to $177, 365 (*p* ≤ 0.02) in the control group. These reduced costs were sustained after 6 months ($223,638 versus $500,343; *p* < 0.03) and at 12 months ($541,378 versus $677,710; *p* < 0.16). Although these findings suggest that costs were decreased, the variable significance levels increase the Type I error rate. Conversely, Copeland and colleagues found no statistically significant differences in cost between the intervention and control groups in their study [39]. As a result, reported cost savings are not consistent when technology is employed for self-management.

#### Quality of Life

Participants in both the intervention and control groups of the trans-telephonic intervention study realized statistically significant changes in quality of life [40]. Conversely, no differences in health-related quality of life were observed in the VA-based telephone intervention study [39]. Quality of life reporting was limited for all studies in that researchers did not report reliability and validity on the instruments used to measure quality of life, and three of the four studies did not report any analysis of data on the Black population sample [37, 39, 40]. It is possible that Blacks might have realized different outcomes when compared to the general population of heart failure patients. Outcomes related to the use of technologically based interventions to promote self-management in Blacks with heart failure might be positive; however, they are currently inconsistent in the existing literature [39].

### Summary and Limitations of Findings

Four very different interventions were tested, and although there are similarities, their findings and their generalizability differ significantly. Because two studies were conducted using small convenience samples and did not include a control group [37, 38••], conclusions from these intervention studies cannot be generalized. One study included randomization and a control group, but participants were randomized based on odd versus even last number of the social security number, which might have contributed to the baseline group differences [39]. Additionally, the short study duration (1 month or 30 days) in three of the four studies does not allow us to draw conclusions regarding whether the results are sustainable over time. Despite these limitations, these four studies suggest significant implications for the future of research regarding using technology to facilitate heart failure self-management in Blacks. Findings generally suggest that the majority of Blacks with heart failure have the required hardware (computer, cell phone) available that will enable them to use these devices to participate in self-management programs and that this participation improves self-confidence and outcomes. However, the patients recruited in these studies might have required training prior to being able to effectively use technological mediums.

## Discussion

Feasibility and use of technology-based interventions to improve self-management is a dominant focus in research to date. In their study, Nundy and colleagues found that only one-third of their participants owned a smartphone [38••], hence smartphone-based interventions might lack feasibility in this patient population. This is consistent with the data reported by the Pew Research Center that indicates that most seniors (age 65 years and older) in the general population use basic cell phones as opposed to smart phones; only 18 % use smartphones when compared to 77 % who use cell phones [44]. In this same report, the lower income, less educated, and older adults in the general population who are over age 80 years were reported to be less likely to use a cell phone or the internet. Though trends in type of phone ownership are changing, these studies indicate that a text messaging program might be more accessible for lower socio-economic groups and older adults who are less likely to use more sophisticated applications. Scherr and colleagues found that using mobile phones as patient terminals for data exchange was effective in reducing hospitalizations in heart failure patients; however, they highlighted the challenge of identifying or designing a user interface that is suitable for older adult use [45]. Kim and colleagues found that text messaging was successful in increasing physical activity among older adult African Americans ages 60 – 85 years [46••]. This suggests that older adult Blacks with heart failure might be willing to use text messaging to aid in heart failure self-management. However, a study conducted by the Pew Research Center indicated that older adults in the general population identified lack of assistance as one barrier to using a cell phone [44]. Once online, 71 % of older adults go online every day [44]. Integrating self-management activities and reminders into an already established daily routine may result in improved adherence, and subsequently improved outcomes. But, an initial time investment for on-boarding might be necessary.

General weaknesses in study design stymie generalizability and suggest opportunities for further research. Existing evidence that evaluates whether technological innovations improve self-management of among Blacks with heart failure is promising, yet inconclusive for several reasons. Beyond the paucity of adequately powered studies, the lack of a control group in two of the studies referenced here make it difficult to infer findings. Second, barriers to technology use in Black heart failure patients, whether similar to or different from that reported in Whites, should be explored. Although 15 patients enrolled in one intervention study, only six remained at study conclusion [38••]. Attrition in technologically based interventions should be explored and methods to promote retention employed. Third, the term “technological innovations” is broad in scope, but incorporates social networking, which is a very popular medium in today’s digital age. Using social networking platforms to facilitate heart failure self-management has not been explored extensively. Social support might result in improved self-care confidence, and subsequently improved self-care [9]. Moreover, social networking might prove beneficial, specifically in the sub-population of Blacks with heart failure, because social support plays a crucial role in the successful management of heart failure in Blacks [18]. Social networking might provide this crucial social support that is necessary for effective heart failure self-management in Blacks because evidence suggests that the source of social support is less important than the actual presence of a confidant with whom to discuss the impact of living with heart failure [18, 22, 47–49]; sharing this experience with others with heart failure who can identify may prove to be more efficacious for heart failure patients when compared to confiding in someone who is not living with the illness [38••]. Dickson and colleagues found that a community-based, in-person program held at a community center was widely acceptable to older adult patients, the majority of which were Black, and that participants were satisfied. This acceptability and satisfaction might be related to the social interaction and support available [50]. Similar social support and interaction might be facilitated using social networking, and could reach those who are homebound due to health status or other issues such as a lack of transportation.

Finally, technology-based research that incorporates caregivers in the deployment of the intervention is needed. Nahm and colleagues noted that participants’ caregivers expressed interest in telemonitoring devices and learning modules, reporting that receiving education via electronic means would assist them in caring for their loved ones [37]. This suggests that technology may be effectively used to assist caregivers of Black heart failure patients with their caregiving responsibilities. Readily accessing information related to heart failure education and management via the internet or receiving this information via text messages might prove beneficial in promoting improved self-management and enhanced outcomes, both to augment the information provided directly to the patient and to aid those who are unable to use the technology themselves. Capitalizing on the opportunity to use caregivers as a resource to aid in self-management using technology among heart failure patients might be a fruitful future direction [48].

## Conclusion

In a review of the literature on telemedicine and remote monitoring of heart failure patients, Anker and colleagues concluded that remote monitoring will become the mainstay of heart failure care and that capitalizing on patients’ self-care abilities is crucial [51•]. Likewise, in their review, Radhakrishnan and colleagues found that telehealth interventions improved self-care abilities and outcomes of heart failure patients. However, they identified the need for future studies with enhanced designs to determine the effectiveness and outcomes that result from patients’ participation in telehealth interventions [52•].

Although the literature specific to Blacks is sparse, findings suggest that using technologically based innovations to facilitate heart failure self-management is beneficial. Interventions such as text messaging programs, online programs for education and monitoring, and social networking platforms provide synchronous and asynchronous education and support that are necessary for successful heart failure self-management and might substantially improve patient and caregiver engagement and clinical outcomes. This might ultimately reduce the heart failure mortality rate and the related disparities among Blacks.
